# Gender Identity, Victimization, and Suicidal Ideation: Comparing Risk Factors Among Transgender and Non‐Transgender Youth

**DOI:** 10.1111/josh.70110

**Published:** 2026-01-04

**Authors:** Whitney DeCamp

**Affiliations:** ^1^ Department of Sociology Western Michigan University Kalamazoo Michigan USA

**Keywords:** bullying, gender, sexual victimization, suicidal ideation, suicide, transgender

## Abstract

**Background:**

Suicidal ideation affects millions of adolescents, with about one‐in‐eight American high school students experiencing suicidal ideation in a given year. Transgender youth and those who have been victimized are at elevated risk.

**Methods:**

Using a sample of over 300,000 American youth, this study examines the prevalence rates of and risk factors associated with suicidal ideation. Differences and similarities between transgender and non‐transgender youth are examined.

**Results:**

Analyses indicate that approximately 18.4% of American high school students have seriously considered suicide within a one‐year period. Transgender youth, however, were 2.6 times as likely (47.1%) to report suicidal ideation than high school students in general. Results of cross‐sectional regression models find that multiple forms of victimization are predictive of suicidal ideation.

**Implications for School Health Policy, Practice, and Equity:**

Intervention strategies, including counseling, are advisable for youth who are at risk for suicidal ideation, such as those who have been victimized or identify as transgender. Supporting the well‐being of youth through systemic policy and practice changes may also lead to improved mental health for those at risk.

**Conclusions:**

Transgender identity is a strong predictor of suicidal ideation even after controlling for other risk factors, such as victimization and substance use. Expanded prevention and intervention efforts are needed to address this growing mental health crisis.

## 1 Introduction

Suicidal ideation, which includes thinking about, considering, or planning suicide [1], is a major concern for youth. Studies of American high school youth (typically aged 14–18) find that approximately 11% to 18% of these adolescents experience suicidal ideation in a given year [2, 3, 4, 5, 6, 7]. Suicide is the third leading cause of death for 15 to 19 year old American youth [8]. Furthermore, research has found that approximately 34%–53% of transgender youth report having seriously considered suicide in the past year [5, 6, 7]. One framework for understanding the higher risk is minority stress theory, which posits that members of stigmatized minority groups suffer under prejudice and discrimination, thus potentially explaining why transgender youth experience more negative outcomes than non‐transgender youth [9, 10]. For example, transgender youth face growing challenges and hostility in the United States; 18 anti‐trans laws were passed in 2021, compared to over 100 in 2025 [11]. New and proposed laws affected the lived experiences of transgender youth, including access to gender affirming care, exposure to harmful conversion therapy, and access to restrooms that match their gender identity. This is particularly problematic for transgender youth given they already experience more discrimination than cisgender youth in schools, such as through health and physical education [12]. The growing politicization of transgender issues and rights may also affect family support and acceptance, which was already precarious and leading to substantial life difficulties among young LGBTQ persons, such as poor mental health and homelessness [13].

Although prior research has found markedly higher prevalence rates for gender‐nonconforming youth in comparison to cisgender youth, the difficulty in obtaining large, representative samples of gender‐nonconforming youth has limited the ability to statistically analyze the correlates and risk factors of suicidal ideation among non‐cisgender youth, particularly with directly comparable models between transgender and non‐transgender youth. Medical research has long relied on the false assumption that using white, male participants in medical trials would provide stability, and the consequence has been findings that may not generalize to the entire population [14, 15]. Likewise, social science research must explore the possibility that risk factors vary across different subpopulations. The present study uses a sample of over 300,000 youth, including over 8000 transgender youth, to explore: (1) the prevalence rate of suicidal ideation in a contemporary, large sample of American high school youth, (2) the risk factors for suicidal ideation, and (3) whether the prevalence rate and risk factors significantly and substantially vary by whether someone is transgender or not.

## 2 Suicidal Ideation Risk Factors

Gender is a well‐established correlate of suicidal ideation. Empirical research consistently finds that female‐identifying individuals are at substantially higher risk for suicidal ideation compared to male‐identifying individuals [16, 17, 18], although one study did not find a significant effect from reported sex among transgender youth [19]. Likewise, research has also identified that those who are not cisgender are at higher risk than cisgender individuals [20, 21, 22, 23, 24].

Victimization is also a common risk factor identified for suicidal ideation and related behaviors. Cyberbullying, for example, has been identified as predictive of suicidal ideation and attempting suicide [25]. Likewise, physical bullying has been found to be associated with self‐injury [26, 27], including among transgender individuals specifically [22]. Sexual assault and other forms of sexual violence have also been found to be risk factors for suicidal ideation [28, 29, 30, 31].

Another theme in suicidal ideation research is substance use and abuse. Research has found mixed evidence on whether tobacco use is a risk factor for suicidal ideation [32, 33], but has more consistently found alcohol use [34, 35, 36] and marijuana use [37, 38] to be risk factors. Evidence is mixed on whether transgender adults are at increased risk for substance use and related disorders [39, 40], but transgender youth are substantially more likely to engage in substance use than non‐transgender youth [41].

## 3 Present Study

As outlined, prior research has identified various risk factors for suicidal ideation, including gender identity. Fewer studies, however, have attempted to determine whether the risk factors vary by gender identity, particularly in making direct comparisons between transgender youth and cisgender youth. Notably, Perez‐Brumer and colleagues [19] provided a valuable examination on this front, but were limited to data from a single state and mostly demographic predictors. Larger samples across multiple states have focused only on prevalence rates and not risk factors [5, 6]. The present study expands on this and other research through the use of a large sample of American high school youth to analyze the prevalence rates and a variety of risk factors in the general population, as well as between transgender and non‐transgender youth.

## 4 Methods

### 4.1 Participants and Procedure

The data used in this study comes from the Youth Risk Behavior Survey (YRBS), which is administered by the Centers for Disease Control and Prevention (CDC) biennially to students in American high schools to track various health indicators among youth [42]. Because only a proportionally small number of youth identify as transgender, data from 4 years of the YRBS (2017, 2019, 2021, and 2023) are pooled together to create a sufficiently large sample. The YRBS data are publicly available on the CDC website [43] and non‐governmental mirrors [44].

A national YRBS dataset is available, but includes only a small portion of cases and would not be a candidate for the present study as it includes only 612 transgender youth. Instead, prior research has combined multiple states/districts to create larger, pooled samples to examine prevalence rates among transgender youth [5, 6]. YRBS data are collected through the CDC's partnership with dozens of other organizations located in various states, but not all of these partners use the standard questionnaire. Data from sites that did not include a question about being transgender are necessarily excluded from this study. Also excluded per CDC guidelines are sites that may contain duplicate participants within the same year. For example, the site for Broward County in Florida is excluded for years in which Florida state data are included because Broward County youth may be represented in both the Broward County data and the Florida data. Combining all sites/years that include the transgender question, except those sites that would be duplicative, results in a dataset containing 429,638 youth. The specific sites used are reported in Appendix A. In total, 26 states have representation in the sample used for this study, either with statewide datasets, or with county or city datasets when statewide data were not available. This includes states with more liberal populations (e.g., California and New York) and states with more conservative populations (e.g., Florida and Oklahoma). States from different regions (e.g., Midwest, Southern, New England) are also represented among the sites. Additional sample demographics are presented in Table 1.

**TABLE 1:** Sample demographics.

Demographic	Unweighted	Weighted
Grade		
9th Grade	28.7%	26.5%
10th Grade	27.1%	25.5%
11th Grade	24.5%	24.2%
12th Grade	19.7%	23.8%
Age		
12 or younger	0.4%	0.4%
13	0.5%	0.5%
14	17.9%	14.3%
15	27.1%	24.8%
16	25.7%	25.5%
17	21.3%	23.6%
18 or older	7.1%	10.9%
Race/ethnicity		
American Indian/Alaska Native	1.0%	1.0%
Asian	6.5%	5.3%
Black/African American	14.3%	15.4%
Hispanic/Latino	17.8%	22.3%
NativeHawaiian/Pacific Islander	2.1%	0.6%
White	51.8%	51.5%
Multiple Races (Non‐Hispanic)	6.5%	4.0%

### 4.2 Instrument

The dependent variable for this study is suicidal ideation, which is measured using the following question: “during the past 12 months, did you ever seriously consider attempting suicide?” Possible responses include “yes” or “no.”

Being transgender is measured using the following question: “Some people describe themselves as transgender when their sex at birth does not match the way they think or feel about their gender. Are you transgender?” The responses offered to participants include: (A) “No, I am not transgender,” (B) “Yes, I am transgender,” (C) “I am not sure if I am transgender,” and (D) “I do not know what this question is asking.” The final response option suggests that the participant does not understand the question, and thus responses do not necessarily indicate transgender identity. The “not sure” response, likewise, could have different meanings, ranging from uncertainty about the term to questioning one's gender. Thus, due to the ambiguity of responses C and D, these latter response options are treated as missing data for the present study. This may result in some gender nonconforming youth being excluded if they answered neither yes nor no to the transgender question. This is unfortunately unavoidable without making assumptions about the entire class of participants who indicated they were “not sure,” which presumably contained a diverse set of reasons for selecting that response option. For clarification, only 2.1% of participants who responded to the transgender question selected C and only 1.8% selected D.

The terms “transgender” and “non‐transgender” are used when describing data and results for this study because the question asked participants whether they are transgender. Some gender non‐conforming youth (e.g., those who identify as nonbinary) may indicate that they are not transgender, but might also not identify as cisgender either if asked. Therefore applying the term “cisgender” to all participants who do not identify as transgender would be inaccurate. This distinction has been used in previous research with similar data limitations [19].

The only measure for sex or gender in the YRBS is from the question, “What is your sex?” Participants are offered only responses of female or male. For a cisgender participant, this is presumably a straightforward question to answer. For a transgender participant, however, the response may be more difficult. On the one hand, the question refers to “sex” rather than “gender,” which suggests it is a biological rather than identity question. On the other hand, it does not refer to “sex assigned at birth” and therefore may provide room for interpretation. This indicator is therefore potentially ambiguous for at least some portion of participants. Nevertheless, the indicator is used as a control variable given its clear connection to the focus of this study. Unfortunately, the YRBS does not currently provide a method for participants to meaningfully identify in their responses as nonbinary. Grade level (9, 10, 11, or 12) is also used as a demographic control variable (responses of “ungraded/other” are considered missing for this study).

A variety of indicators are used to capture different forms of victimization. These questions include: “during the past 12 months, have you ever been electronically bullied? (Count being bullied through texting, Instagram, Facebook, or other social media)” [no/yes]; “during the past 12 months, have you ever been bullied on school property?” [no/yes]; “during the past 30 days, on how many days did you not go to school because you felt you would be unsafe at school or on your way to or from school?” [five‐point ordinal scale]; “during the past 12 months, how many times has someone threatened or injured you with a weapon such as a gun, knife, or club on school property?” [eight point ordinal scale]; “during the past 12 months, how many times did anyone force you to do sexual things that you did not want to do? (Count such things as kissing, touching, or being physically forced to have sexual intercourse)” [five‐point ordinal scale]; and “have you ever been physically forced to have sexual intercourse when you did not want to?” [no/yes].

Indicators are also used to capture substance use. Participants were asked how often they used cigarettes, alcohol, and marijuana in the past 30 days. For each substance, participants were presented with an ordinal scale for the amount of use. Descriptive statistics for all variables are presented in Table 2.

**TABLE 2:** Descriptive statistics (unweighted).

Variable	Min	Max	*N*	Percent	Non‐Trans, percent	Trans, percent	Sig.
Considered suicide	0	1	347,284	18.2	16.8	51.2	**
Transgender	0	1	398,543	2.6	0.0	100.0	—
Sex (female)	0	1	420,107	50.3	50.5	55.4	**
Grade level	1	4	419,354				**
9th grade				28.7	28.6	28.1	—
10th grade				27.1	27.2	27.4	—
11th grade				24.5	24.5	24.2	—
12th grade				19.7	19.7	20.2	—
Digital bullying	0	1	421,148	15.4	14.6	34.0	**
Bullied at school	0	1	344,901	18.0	16.9	40.3	**
Safety concerns	1	5	366,532				**
0 days				91.1	92.1	75.1	—
1 day				4.5	4.3	8.3	—
2 or 3 days				2.5	2.2	7.0	—
4 or 5 days				0.6	0.5	2.2	—
6 or more days				1.2	0.9	7.3	—
Threatened at school	1	8	372,839				**
0 times				93.0	93.9	78.7	—
1 time				3.2	3.0	6.8	—
2 or 3 times				1.9	1.7	4.6	—
4 or 5 times				0.6	0.5	2.1	—
6 or 7 times				0.3	0.2	1.2	—
8 or 9 times				0.1	0.1	0.6	—
10 or 11 times				0.1	0.0	0.4	—
12 or more times				0.8	0.6	5.6	—
Forced sexual acts	1	5	177,637				**
0 times				89.1	90.2	70.6	—
1 time				5.3	5.0	9.0	—
2 or 3 times				3.3	3.0	7.9	—
4 or 5 times				0.8	0.7	3.7	—
6 or more times				1.5	1.1	8.8	—
Forced intercourse	0	1	222,986	8.3	7.4	26.0	**
Cigarette use	1	7	415,262				**
0 days				94.7	95.4	82.8	—
1 or 2 days				2.1	2.0	4.5	—
3 to 5 days				0.8	0.7	2.3	—
6 to 9 days				0.5	0.4	1.5	—
10 to 19 days				0.5	0.4	1.5	—
20 to 29 days				0.3	0.3	0.8	—
All 30 days				1.0	0.8	6.7	—
Alcohol use	1	7	398,809				**
0 days				76.1	76.2	70.1	—
1 or 2 days				13.1	13.3	12.5	—
3 to 5 days				5.5	5.6	5.3	—
6 to 9 days				2.8	2.7	3.0	—
10 to 19 days				1.4	1.4	2.0	—
20 to 29 days				0.4	0.4	0.8	—
All 30 days				0.7	0.5	6.3	—
Marijuana use	1	6	410,635				**
0 times				81.9	82.3	73.5	—
1 or 2 times				6.4	6.4	7.7	—
3 to 9 times				4.4	4.4	5.3	—
10 to 19 times				2.3	2.2	2.7	—
20 to 39 times				1.7	1.7	2.3	—
40 or more times				3.2	3.0	8.5	—
Survey year	1	4	423,275				**
2017 (1)				27.2	26.8	22.6	—
2019 (2)				28.0	28.6	17.4	—
2021 (3)				21.5	21.6	26.2	—
2023 (4)				23.3	23.0	33.8	—

** *p* < 0.01 (chi‐square difference test for non‐transgender vs. transgender).

### 4.3 Data Analysis

The sampling procedures for the YRBS vary between sites and may include oversampling some populations. Case weights are calculated by the CDC in order to ensure samples are representative of the populations from which they were drawn. Some sites provide data across multiple years, and the case weights for those sites are divided by the number of years used for that site, as recommended by CDC guidelines for using data pooled across years [45]. As an example, data are available from Wisconsin for all 4 years, but data from Utah are only available for 1 year. Applying the case weights as originally provided would result in Wisconsinites being four times as represented as Utahns (in addition to whatever population size difference should be present). In order to prevent the sample from being overly representative of Wisconsinites, the case weights for these participants are divided by four. As an exception, the original case weights are used for year‐specific analyses, as adjusting for multiple years is not applicable in those analyses.

The first series of analyses involves examining suicidal ideation prevalence rates. Statistical significance of differences in prevalence rates across groups or years was estimated using Pearson chi‐square tests. This is followed with a series of logistic regression models predicting suicidal ideation, including in the general youth population, among transgender youth, and among non‐transgender youth. Although these models are illustrative of effect sizes among different groups, the significance indicators are not directly comparable given the statistical power differential between the transgender and non‐transgender sub‐samples. Therefore, additional models are estimated with heterogeneous choice models using the oglm procedure [46, 47], which allows for interaction effects to identify when there is sufficient statistical evidence to conclude that an effect size differs between groups. All regression models are estimated using Stata 18.

Prior to analysis, missing data must be handled with care. Data may be missing for a number of reasons, including: a participant leaving a question unanswered, unreadable, or otherwise invalid; a question being excluded from the questionnaire for a particular site; or the response having been recoded as missing (e.g., as described in the variable section for grade level and transgender). Cases with severe missing data issues (i.e., missing on more than one‐third of the variables used) are removed listwise, resulting in a sample size of 423,275 (down 1.5%). For the remaining cases, missing data on any variable except transgender and suicidal ideation was handled using multiple imputation in Stata 18 with a chained model using logistic regression for dichotomous variables and ordered logistic regression for ordinal variables. Five sets of imputations were created through 50 model iterations. Diagnostics of the variables' means and standard deviations across imputations and non‐imputed data indicated only trivial (non‐significant) variations. To identify missing data by variable, the case numbers reported in Table 2 reflect the valid responses prior to imputation. The final sample size for the regression models, after excluding cases missing on transgender and suicidal ideation, is 326,398 youth.

## 5 Results

Univariate analyses of the data with case weights applied indicate that approximately 18.4% (±0.1% with a 95% confidence interval) of American high school youth have considered suicide within the past 12 months. Comparing the prevalence rate for suicidal ideation by transgender identity, however, reveals a substantial disparity, with approximately 47.1% (±1.1%) of transgender youth having considered suicide in the past 12 months, compared to 17.1% (±0.1%) of non‐transgender youth. The difference between non‐transgender and transgender youth in suicidal ideation is statistically significant (*p* < 0.001), and there are also significant differences between non‐transgender and transgender youth on all other variables.

Examining the prevalence rates by years suggests changes over time. Among the general population the suicidal ideation rates are 17.3% for 2017, 16.6% for 2019, 19.1% for 2021, and 19.0% for 2023. The increase from 2017 to 2023 is statistically significant (*p* < 0.001). Among transgender youth the suicidal ideation rates are 43.2% for 2017, 45.2% for 2019, 51.6% for 2021, and 48.5% for 2023. The increase from 2017 to 2023 is statistically significant (*p* < 0.001). To control for this upward trend, regression models include a control variable for the survey year.

Results for logistic regression models using the full sample are presented in Table 3. The first model includes only transgender identity and sex to establish baseline effects for gender. Transgender youth are significantly more likely to report having considered suicide in the past year compared to those who are not transgender (OR = 4.298). Likewise, youth who report being female are significantly more likely to report having considered suicide than those who report being male (OR = 2.145).

**TABLE 3:** Logit models predicting suicidal ideation.

	Model 1					Model 2				
	*b*	SE	*β*	OR		*b*	SE	*β*	OR	
Transgender	1.458	0.069	0.231	4.298	**	0.978	0.080	0.155	2.658	**
Sex (female)	0.763	0.027	0.382	2.145	**	0.605	0.030	0.302	1.831	**
Grade level	—	—	—	—		−0.042	0.013	−0.046	0.959	**
Digital bullying	—	—	—	—		0.617	0.041	0.224	1.854	**
Bullied at school	—	—	—	—		0.690	0.040	0.266	1.993	**
Safety concerns	—	—	—	—		0.110	0.024	0.066	1.116	**
Threatened at school	—	—	—	—		0.078	0.024	0.063	1.081	**
Forced sexual acts	—	—	—	—		0.256	0.025	0.177	1.292	**
Forced intercourse	—	—	—	—		0.681	0.051	0.189	1.976	**
Cigarette use	—	—	—	—		0.046	0.022	0.037	1.047	*
Alcohol use	—	—	—	—		0.097	0.017	0.099	1.102	**
Marijuana use	—	—	—	—		0.181	0.013	0.210	1.198	**
Survey year	—	—	—	—		0.067	0.014	0.075	1.069	**
Model statistics	McFadden *R* ^2^ = 0.035 chi‐square = 1229.94 df = 2 *n* = 326,398 *p* < 0.001					McFadden *R* ^2^ = 0.133 chi‐square = 3847.58 df = 13 *n* = 326,398 *p* < 0.001				

*Note*: *p < 0.05; **p < 0.01. *b* = unstandardized coefficient; SE = standard error of *b*; β = standardized coefficient; OR = odds ratio.

The second model in Table 3 provides a full model with all predictors. As with the initial model, transgender youth (OR = 2.658) and female youth (OR = 1.831) are significantly more likely to have considered suicide than their non‐transgender and male counterparts respectively. All six measures of victimization are significantly associated with higher risk of considering suicide, including digital bullying (OR = 1.854), bullying at school (OR = 1.993), safety concerns (OR = 1.116), threats at school (OR = 1.081), forced sexual acts (OR = 1.292), and forced intercourse (OR = 1.976). All three forms of substance use in the model are also associated with higher risk of considering suicide, including cigarettes (OR = 1.047), alcohol (OR = 1.102), and marijuana (OR = 1.198). Consistent with the prevalence rates observed as increasing over time, the year in which the data were collected is also significantly associated with considering suicide (OR = 1.069).

Separate analyses of non‐transgender and transgender youth are presented in Table 4. The model for non‐transgender youth closely replicates the findings from the general population, which is not surprising given that about 97% of the overall sample is non‐transgender. The model for transgender youth has several notable differences. In particular, the effects from grade level, safety concerns, threats at school, forced sexual acts, substance use, and survey year are not statistically significant. The other effects remain significant, though they vary in strength, ranging from a 36% decrease in effect size for forced intercourse to a 27% increase in effect size for sex. Caution should be taken in inferring any meaningful differences based on differences in significance levels between the non‐transgender and transgender models, as the massive difference in the sample sizes also results in differences in statistical power that may cause similarly sized effects to have differing significant levels.

**TABLE 4:** Logit models predicting suicidal ideation by transgender.

	Non‐transgender youth					Transgender youth						
	*b*	SE	β	OR		*b*	SE	β	OR		Δ	Δ%
Sex (female)	0.588	0.030	0.294	1.801	**	0.749	0.153	0.375	2.115	**	−0.080	27%
Grade level	−0.043	0.014	−0.046	0.958	**	−0.037	0.066	−0.040	0.964		−0.006	−13%
Digital bullying	0.624	0.042	0.226	1.866	**	0.438	0.189	0.159	1.550	*	0.067	−30%
Bullied at school	0.680	0.041	0.262	1.974	**	0.794	0.165	0.306	2.211	**	−0.044	17%
Safety concerns	0.119	0.024	0.072	1.127	**	0.105	0.083	0.063	1.110		0.009	−12%
Threatened at school	0.103	0.024	0.083	1.108	**	−0.017	0.069	−0.013	0.984		0.096	−116%
Forced sexual acts	0.268	0.025	0.186	1.307	**	0.167	0.087	0.116	1.181		0.070	−38%
Forced intercourse	0.695	0.055	0.193	2.005	**	0.443	0.215	0.123	1.558	*	0.070	−36%
Cigarette use	0.066	0.023	0.052	1.068	**	0.036	0.076	0.028	1.036		0.024	−46%
Alcohol use	0.111	0.018	0.113	1.117	**	−0.053	0.076	−0.054	0.948		0.167	−148%
Marijuana use	0.185	0.013	0.214	1.203	**	0.051	0.068	0.059	1.052		0.156	−73%
Survey year	0.065	0.014	0.073	1.067	**	0.090	0.070	0.100	1.094		−0.027	38%
Model statistics	McFadden *R* ^2^ = 0.125 chi‐square = 3608.18 df = 12 *n* = 318,390 *p* < 0.001					McFadden *R* ^2^ = 0.097 chi‐square = 125.68 df = 12 *n* = 8008 *p* < 0.001						

*Note*: *p < 0.05; **p < 0.01. *b* = unstandardized coefficient; SE = standard error of *b*; β = standardized coefficient; OR = odds ratio. Δ = Difference in *β* between Model 1 and Model 2; Δ% = Δ as a percent of Model 1 *β*.

The final models (see Table 5) use interaction effects to allow for direct comparisons between non‐transgender and transgender youth. This approach uses a pooled model and employs interaction effects to estimate differences, thus the significance tests directly assess the differences between transgender and non‐transgender youth. The first model largely replicates the original full model (Table 3, Model 2), although the estimates have trivial variances from the prior model due to the different estimation technique. The second model introduces interaction effects for each of the substantive effects. The interaction effects indicate that these effects are less powerful for transgender youth, although few of these differences are statistically significant. Specifically, only alcohol use (*p* = 0.015) and marijuana use (*p* = 0.008) are significantly different by transgender identity, with the effects reduced substantially for transgender youth compared to non‐transgender youth. For context, transgender youth are significantly (*p* < 0.001) more likely than non‐transgender youth to use alcohol (29.7% vs. 23.8%) and marijuana (25.4% vs. 17.1%). Of particular note is the transgender effect size in this final model. Whereas transgender identity did not stand out as an especially powerful effect in prior models, here transgender is the most powerful effect (*β* = 0.340). This suggests that failing to control for interaction effects between transgender identity and other predictors may obscure the true impact of being transgender.

**TABLE 5:** OLGM models predicting suicidal ideation.

	Model 1				Model 2			
	*b*	SE	*β*		*b*	SE	*β*	
Transgender	0.836	0.131	0.132	**	2.146	0.208	0.340	**
Sex (female)	0.601	0.030	0.300	**	0.589	0.030	0.294	**
Grade level	−0.042	0.014	−0.046	**	−0.042	0.013	−0.045	**
Digital bullying	0.626	0.042	0.227	**	0.624	0.042	0.226	**
Bullied at school	0.694	0.041	0.267	**	0.680	0.041	0.262	**
Safety concerns	0.118	0.024	0.071	**	0.119	0.024	0.072	**
Threatened at school	0.093	0.024	0.075	**	0.103	0.024	0.083	**
Forced sexual acts	0.267	0.025	0.185	**	0.268	0.025	0.186	**
Forced intercourse	0.696	0.053	0.193	**	0.695	0.055	0.193	**
Cigarette use	0.058	0.023	0.046	*	0.066	0.023	0.052	**
Alcohol use	0.105	0.018	0.106	**	0.111	0.018	0.113	**
Marijuana use	0.184	0.013	0.213	**	0.185	0.013	0.214	**
Survey year	0.067	0.014	0.075	**	0.066	0.014	0.073	**
Trans * electronic bullying	—	—	—		−0.278	0.174	−0.026	
Trans * bullied at school	—	—	—		−0.056	0.177	−0.006	
Trans * safety concerns	—	—	—		−0.037	0.071	−0.011	
Trans * threatened at school	—	—	—		−0.116	0.059	−0.046	
Trans * forced sexual acts	—	—	—		−0.136	0.078	−0.044	
Trans * forced intercourse	—	—	—		−0.344	0.202	−0.026	
Trans * cigarette use	—	—	—		−0.039	0.065	−0.014	
Trans * alcohol use	—	—	—		−0.152	0.062	−0.061	*
Trans * marijuana use	—	—	—		−0.144	0.055	−0.055	**
Model statistics	Trans ς = 0.547 *f* = 9415.8 df = 14 *n* = 326,398 *p* < 0.001				Trans ς = −0.238 *f* = 11,560.2 df = 23 *n* = 326,398 *p* < 0.001			

*Note*: *p < 0.05; **p < 0.01. *b* = unstandardized coefficient; SE = standard error of *b*; *β* = standardized coefficient.

## 6 Discussion

Suicidal ideation is a serious concern for today's youth and young adults, and the analyses presented here find a prevalence rate for seriously considering suicide within the past year of approximately 18.4% among American high school students. Among transgender students, the prevalence rate is 47.1%, which is similar to recent research using high school data [6, 7]. Further, the trend analysis indicates that the prevalence rate increased over the years included in this study. For transgender youth, this may be reflective of the increasing political and legal hostility towards transgender persons in the United States [11], which may also affect the negative treatment many experience at school and in their familial relations, which is already substantial [12, 13]. Notably, however, the increasing prevalence rate is found for both the general population and transgender youth specifically, and in approximately similar proportions of 9.8% and 12.3% increases, respectively. Nevertheless, the net impact of these increases is much more pronounced for transgender youth given their substantially higher baseline risk.

All risk factors examined here are significant predictors of suicidal ideation. Indicators of bullying are particularly influential risk factors, with victims of digital bullying 1.9 times as likely to consider suicide and victims of school‐based bullying 2.0 times as likely compared to youth who are not bullied. Although the exact ratio varies slightly, this general effect was found for both non‐transgender and transgender youth. This extends prior findings [25, 26, 27] to transgender youth in this sample. Similarly, sexual victimization is a significant predictor of considering suicide, with victims of forced intercourse being about 2.0 times as likely to consider suicide compared to those without such victimization. Significant effects were found regardless of transgender identity, supporting previous findings in general population studies [28, 29, 30, 31].

The most predictive indicators, however, are those of transgender identity and sex. Consistent with prior research [14, 18], the regression model indicates that those who are female are 1.8 times as likely to experience suicidal ideation than those who are male, and those who are transgender are 2.7 times as likely to experience suicidal ideation than those who are non‐transgender. The effect from transgender is particularly powerful in the final model, which controlled for interaction effects between transgender and other risk factors, suggesting that the true impact of transgender identity can be obscured when interaction effects are not included in the model. Further, although several predictors were not significant in the initial transgender model, the final model that directly compared effects by transgender identity among the entire sample suggests that there is not sufficient evidence to conclude that the effects for transgender youth differ from effects for non‐transgender youth for most risk factors. Specifically, only the effects of alcohol use and marijuana use were significantly different (reduced) for transgender youth. The reason for these reduced effects is unclear—it may, for example, be a competing coping mechanism for transgender youth. Transgender youth are 25% more likely to use alcohol and 49% more likely to use marijuana than non‐transgender youth, so some of the reduced effect may be because substance use is more normative in this population and therefore less likely to be an indicator of distress. Future research should further explore these relationships.

## 7 Implications for School Health Policy, Practice, and Equity

Identifying youth at risk for suicidal ideation and suicidal behavior is of substantial value considering the high proportion of youth deaths that occur through suicide. These findings lend further support for known risk factors of suicidal thoughts and behavior, including bullying, sexual victimization, and substance use. Importantly, however, transgender youth are found to be at significantly higher risk even after controlling for these risk factors. In other words, transgender youth are at elevated risk for suicidal ideation merely by being transgender, even if they are not experiencing bullying or other forms of victimization. It is therefore advisable to consider youth with any of these risk factors, even in isolation, as potentially benefiting from services and other forms of support.

Counseling and other intervention strategies for youth who are experiencing or have experienced victimization are advisable, but with the additional knowledge that transgender youth are at a baseline elevated risk and may be especially in need of support. Beyond intervention strategies, others have already called for system change to support the well‐being of transgender youth [12], and this research adds further support for bullying and victimization prevention as one area that would benefit from such change. The CDC's “What Works in Schools” program recommends a focus on improving connectedness through training teachers to support mental and behavior health of their students, offering youth development and mentoring programs, and adopting policies for anti‐harassment, safe spaces, and other inclusive practices [48]. This research finds that various forms of victimization compound the already high risk for suicidal ideation among transgender youth, supporting minority stress theory and the proposition that the stress these youth experience is associated with substantial risk. Efforts by schools to create an inclusive and safe environment would benefit transgender students, as well as students in general [48, 49].

## 8 Limitations

The data and analyses in the regression models are cross‐sectional, and therefore it is important to avoid inferring causality. The models and relationships estimated here may help to identify individuals at increased risk, but do not establish that any of these risk factors have a direct, causal effect on suicidal ideation. Nevertheless, the consistent significance of the effects suggests that the potential for identifying high‐risk individuals through these indicators may be valuable in addressing one of the leading causes of death in these age groups. Another limitation of note is that the data only include youth from 26 of the 50 states, some of which are represented only partially through county or city data. Importantly, however, the collection of states represented does include both politically conservative and liberal states, as well as states from all geographic regions of the country. Some states have or are creating environments that are more hostile to transgender youth, but that does not seem to be related to participating sites in the 2017–2023 data. Whether the rapidly evolving political and legal hostility in some areas is further affecting suicidal ideation by transgender identity is another potential line of inquiry for future research.

## 9 Conclusions

Prior research has indicated that transgender youth are at particularly high risk for suicide and suicidal ideation. This study lends further support to that conclusion, finding that 47% of transgender American high school students have seriously considered suicide within the past year. Other risk factors, including bullying and sexual victimization, are similar between transgender and non‐transgender youth, although the effects of substance use are not as influential for transgender youth. Overall, this study affirms that transgender youth are at substantially higher risk for suicidal ideation, and support mechanisms to address this directly, as well as the underlying causes, are urgently needed. The list of potential actions that could be taken to address these needs is lengthy, but the actions most directly related to the risk factors examined here are those that might reduce victimization, including bullying. The current environmental changes risk increasing potential for bullying, such as through restricting restroom access, removing accommodations, requiring teachers to use pronouns that may not reflect those that are preferred, denying the existence of gender outside a strict binary, and so on. In contrast, an environment free from these hostilities and one that fosters supportive relationships with teachers may reduce bullying and other forms of victimization. Although school victimization is not the only factor associated with suicidal ideation for transgender youth, it is a substantial one and this research suggests that working to make our schools a safe and accepting environment would have a beneficial impact on mental health and well‐being.

## Ethics Statement

The author has nothing to report.

## Conflicts of Interest

The author declares no conflicts of interest.

## Data Availability

The data that support the findings of this study are openly available in the Centers for Disease Control and Prevention at https://www.cdc.gov/yrbs/index.html.

## Appendix A Appendix: YRBSS Sites Included in the Present Study

**Table:**

Site	Years	Site name
CE	2	Cleveland, OH
CH	2	Chicago, IL
CO	2	Colorado
DE	1	Delaware
FL	2	Florida
FT	1	Broward County, FL
HI	4	Hawaii
IA	1	Iowa
IL	1	Illinois
IN	1	Indiana
KY	1	Kentucky
LO	4	Los Angeles, CA
MD	4	Maryland
ME	2	Maine
MI	4	Michigan
MS	1	Mississippi
ND	2	North Dakota
NE	1	Nebraska
NH	1	New Hampshire
NJ	3	New Jersey
NV	3	Nevada
NY	1	New York
NYA	2	New York (excl. NYC)
NYC	2	New York City, NY
OK	1	Oklahoma
PA	3	Pennsylvania
PO	3	Portland, OR
RI	2	Rhode Island
SA	4	San Diego, CA
SE	3	Seattle, WA
SF	2	San Francisco, CA
UT	1	Utah
VA	3	Virginia
VT	4	Vermont
WI	4	Wisconsin
